# Wireless Patrol Sign-In System with Mental Fatigue Detection

**DOI:** 10.1155/2018/6419064

**Published:** 2018-11-12

**Authors:** Kang-Ming Chang, Hao-Chen Xu, Congo Tak-Shing Ching, Shing-Hong Liu

**Affiliations:** ^1^Department of Photonics and Communication Engineering, Asia University, Taichung, Taiwan; ^2^Department of Medical Research, China Medical University Hospital, China Medical University, Taichung, Taiwan; ^3^Department of Electrical Engineering, National Changhua University of Education, Taiwan; ^4^Graduate Institute of Biomedical Engineering, National Chung Hsing University, Taichung, Taiwan; ^5^Department of Computer Science and Information Engineering, Chaoyang University of Technology, 168, Jifeng E. Rd., Wufeng District, Taichung 41349, Taiwan

## Abstract

Current sign-in methods of patrolling security guards mainly comprise signature, image identification, and fingerprint identification; notably, none of these methods indicate the physical and mental conditions of such guards. In particular, when patrolling security guards perform their duties consecutively for a long period of time, adequate attention should be directed toward their levels of mental fatigue. When a handwriting sign-in system is adopted, security guards may not record their sign-in time accurately, or they may fake signatures during long shifts. In addition, image identification systems cannot comprehensively reflect the physical and mental statuses of on-duty security guards, particularly their levels of fatigue. Monitor fatigue in patrolling security guards is important to avoid burnout and stress in the workplace. Therefore, in this study, a patrolling sign-in system that integrates physiological signals and images was designed. A thermometer, hand dynamometer, and electromyography sensor were combined to measure physiological signals. Results showed that hand grip strength and the median frequency of electromyography signals gradually reduced when muscle fatigue occurred. The system determined whether a security guard had signed in punctually and whether this person should stay on duty. Overall, this system was verified to operate effectively, and it is therefore applicable for monitoring the sign-in of patrolling security guards who work long shifts. This case series study proposed a conceptual prototype of the system; large-scale testing should be performed in subsequent research.

## 1. Introduction

Human-conducted patrol, such as that performed by police, is an important topic that has been thoroughly investigated. Although robots [1], artificial intelligence technology [2], and cameras [3] have been widely adopted for specific security tasks, human patrol remains indispensable. Security patrolling mainly involves signing an attendance sheet and observing the surrounding environment to detect abnormalities.

Mental fatigue of security guards is a key concern. Previous studies on the mental fatigue of security guards have typically focused on driver fatigue [4]. For example, Lee et al. analyzed drivers' fatigue with an automatic diagnosis system that collected health information from the drivers [5]. Zhang et al. developed a fatigue detection system for high-speed train drivers that comprised a wireless wearable electroencephalograph and support vector machine [6]. Body fatigue can also be detected by examining physiological parameters, such as saccadic velocity [7]. For example, Mandal et al. assessed drivers' fatigue by studying face and eye openness [8].

Indeed, physiological signals have been widely adopted to detect fatigue; in particular, these signals have proven effective in detecting muscle fatigue according to a person's muscle contractions. There are several questionnaires developed to measure muscle fatigue [9, 10], and there is high correlation between these questionnaires and EMG-derived features [11]. Some researchers have explored the muscle and shoulder posture discomfort experienced by patrolling police and their relationship with fatigue [12]. A physiological-based method was also used to detect helicopter pilots' fatigue by monitoring their neck and shoulder muscle activity [13]. Elsewhere, Zużewicz et al. used heartbeats and electromyography (EMG) signals to study the physical responses of car drivers. Their results showed that EMG signal amplitude is very sensitive to such responses [14]. The median frequencies of EMG signals are often adopted as a key indicator of muscle fatigue. For instance, Dong et al. examined wireless EMG signals that were collected using a fatigue-tracking system [15]. Liu et al. used ensemble empirical mode decomposition to analyze high-frequency EMG signals and thereby determine muscle fatigue. Notably, the ensemble empirical mode decomposition method is superior to traditional EMG signal detecting methods and therefore provides a better understanding of muscle fatigue [16]. González-Izal examined muscle fatigue associated with prolonged standing in the workplace by EMG, and the results showed that there was 20% decrease in median frequency after two hours of working. They also found that there were averagely 15% decrease in median frequency during a dynamic fatiguing protocol [17]. Therefore decrease of 20% of median frequency can be used as primary muscle fatigue index.

In addition to detecting mental fatigue, biomedical signal measurements such as body temperature and grip strength can be used to determine whether an on-duty security guard is experiencing hypothermia or muscle fatigue; this allows suitable response measures to be implemented. Moreover, if sign-in systems can be integrated with distance monitoring, user convenience can be improved [18]. In the present study, distance monitoring was combined with muscle fatigue and body temperature detection measures to create a smart sign-in system. A graphical software development program (LabVIEW) was used to write the programming code, which could analyze the digital signals that extractors sent to a computer and elucidate their characteristics. Subsequently, temperature, grip strength, and EMG measurements were compiled, stored, and sent to the server to build a database. Furthermore, a user-friendly interface was developed through which values could be sent back to the monitoring end to reveal the temperature, grip strength, and EMG measurements of each security guard. This enabled the monitor to identify whether a security guard signs in punctually, and whether he or she is suitable for duty. These functions facilitate the administration of appropriate care and the maintenance of operational procedures.

## 2. Materials and Methods

### 2.1. System Implementation

The proposed system comprises the Vernier sensor system (Vernier Software and Technology) and signal extracting and monitoring systems, the latter two of which were written using LabVIEW. The Vernier sensor system consists of a charge-coupled device (CCD), a hand dynamometer, temperature sensors, and an EMG. The specifications are as follows:

- Temperature sensors (STS-BTA): range of measurable temperature −25 to 125°C
- Hand dynamometer (HD-BTA): this measures grip and pinch strength; resolution: 0.2141 N; operational range: 0–600 N
- Electromyography (EKG-BTA): this measures EMG values before and after grip strength detection through electrodes attached to the wrist
- CCD (Model: acA1300-30 gm): resolution: 1296 × 966

**Figure 1:**
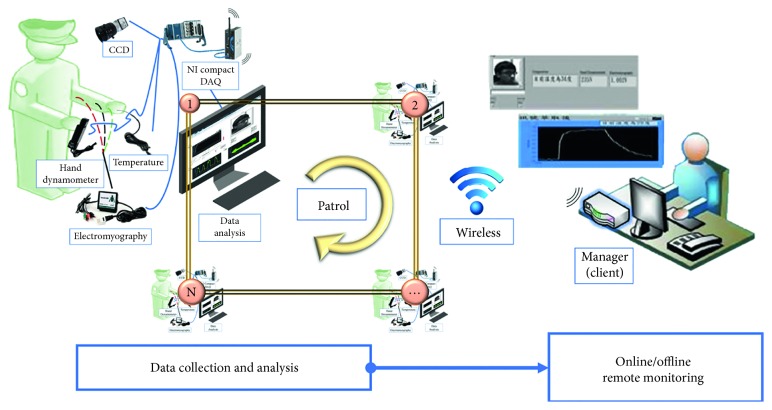
Framework of the wireless patrol sign-in monitoring system.

The proposed system also uses an NI Compact DAQ-9184 as its analog-digital converter. The system collected four physiological signals through a set of sensor and data extraction sites that were installed at every sign-in station on the studied patrol path. Wireless routers were used to send signals containing the extracted information to fix Internet protocol addresses at the back end for an administrator to review, either in real time or at a subsequent time.  Figure 1 outlines the system framework.

### 2.2. Feature Extraction and System Interface

**Figure 2:**
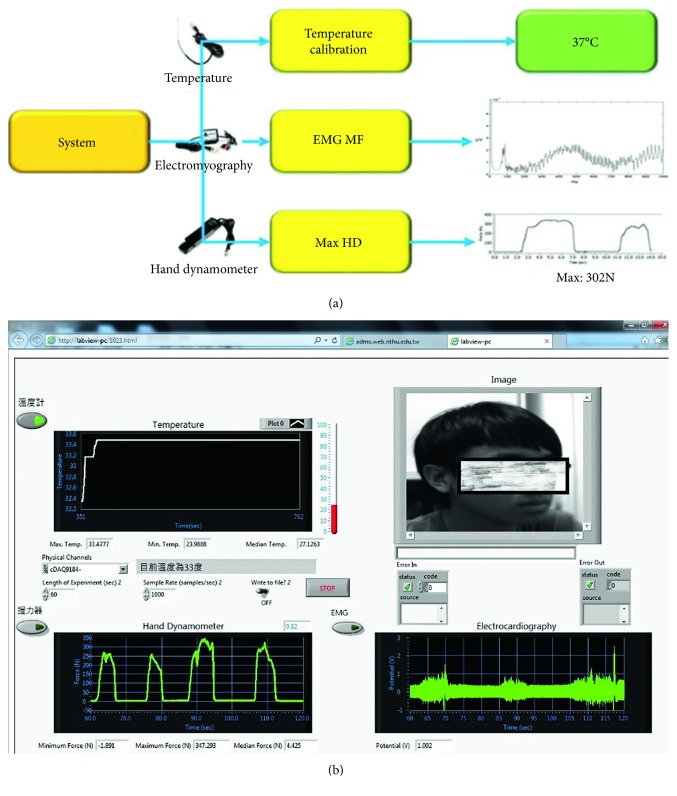
(a) Diagram of the system interface. (b) Back-end observation interface of the system.

Figure 2(a) presents a diagram of the system interface and shows how the stable temperature measurements, EMG signals, and maximum hand grip strength are observed at the back end.  Figure 2(b) shows the back-end observation interface of the system.

Three biosignals, temperature, hand grip strength, and muscle contraction are recorded every 30 minutes. Security guards were also asked to face the camera at each measurement station to have their pictures taken and uploaded to the system. First recorded biosignal is temperature. A mercury thermometer was adopted as the standard measurement tool, although both an NI device and mercury thermometer were used to measure five points under different water temperatures. The analysis results showed linear temperature variations. Using the mercury thermometer measurements as the standard, a linear conversion equation was derived, through which the temperature measurements obtained using the NI device were converted to those acquired using the mercury thermometer. Notably, temperatures were found to change from ambient temperature to body temperature; however, the system only sends stable body temperature measurements to the back end to prevent data excess. To determine temperature stability, a buffer was added to the program. Temperature that varied <0.01°C before and after extraction was deemed stable. These criteria of temperature calibration and temperature stability were all directly written into the program code. This enabled the system to accurately measure temperature signals and output stable body temperature measurements through temperature sensors.

Second recorded biosignal is hand strength. Each subject is required to grip hand dynamometer 2–5 trials. Each grip strength test trial was performed for 2–5 s, and each trial interval is 1 to 2 s. Following grip signals are processed with window size being 5 seconds. Maximum value of each trial within window is extracted. Maximum value of these trials is treated as grip strength. Third recorded biosignal is muscle contraction. When the subject grips the dynamometer, electromyography (EMG) is also recorded at the same time. The recorded EMG data were imported to a Hamming window with 1024 points. Notably, 50% overlapping was conducted to convert the time domain EMG data to frequency domain spectrums with a 1024-Hz fast Fourier-transform. Median frequency of each power spectrum distribution is also estimated. Average median frequency of each trial is used as the following muscle fatigue feature. For the whole night recording, the first recorded median frequency is treated as resting state. The following median frequency derived from the other time session is divided by the first recorded median frequency. The time-varying median frequency is demonstrated as percentage scale.

### 2.3. Experimental Process

**Figure 3:**
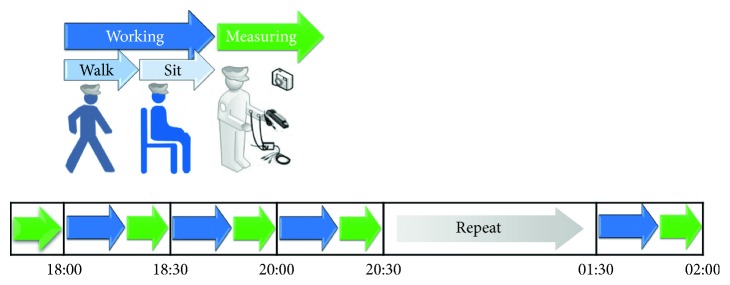
Experimental process of system overnight testing.

To test whether the system could be applied to actual patrolling, an experiment was designed and performed. One man and one woman (both 22-year olds) were recruited as the respondents. The ambient temperature of the patrol area was 14°C, and the measuring time was 8 h (from 6:00 pm to 2:00 am), which is similar to a regular nightshift security guard schedule. Biomedical signals (i.e., body temperature, hand grip strength, and EMG signals) and photographs of the respondents were collected every 30 min (15 min of measuring, 10 min walking, and 5 min of rest) and input into the system for analysis. In between the data collection periods, the respondents were walking freely throughout the designated area.  Figure 3 presents a diagram of the experimental process.

## 3. Results

**Figure 4:**
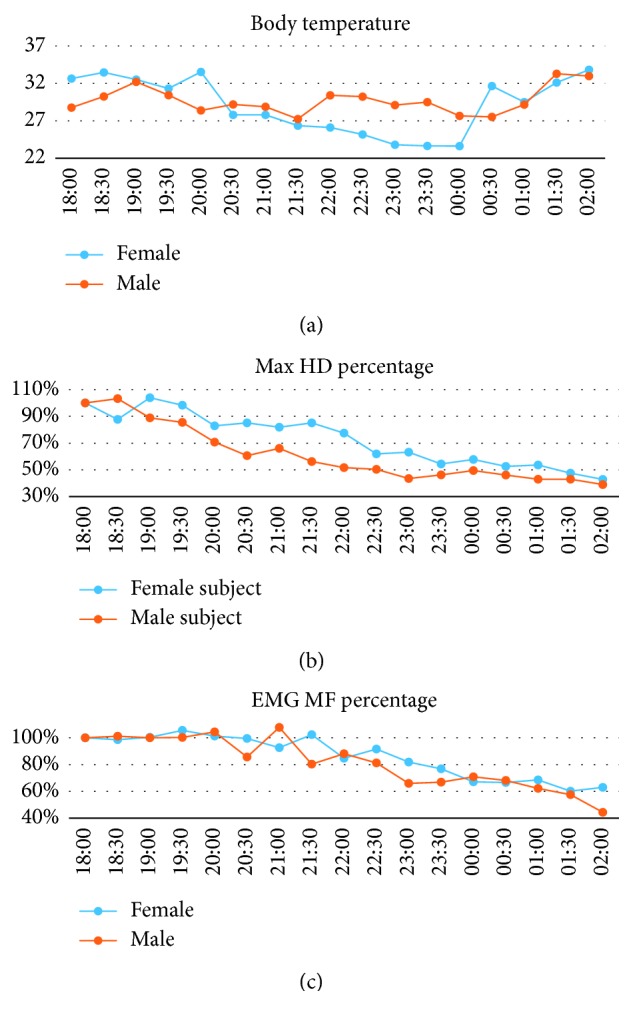
System output of the 8-hour simulated patrol test.

Figure 4 displays the temperature, maximum grip strength, and median frequency of muscle contraction values obtained from the respondents during 8 h of simulated patrolling and testing. According to Figure 4, the respondents' body temperatures ranged from 23 to 34°C. Notably, the male respondent and female respondent demonstrated different maximum grip strengths, which ranged between 140 and 180 and between 60 and 70, respectively. Percentages of maximum grip strengths are decreased from original grip strength to 40%–45%, as shown in Figure 4. Additionally, decreased median frequencies were observed between 9:30 pm and 2:00 am in Subject 1, and between 8:00 pm and 2:00 am in Subject 2 (Figure 4). The median frequency ranged from 240 Hz to 140 Hz and from 340 Hz to 140 Hz for Subject 1 and Subject 2, respectively. Ratio of median frequency is decreased to 80% around 22:30 to 23:00 and is decreased to 60% around 1:30 am, seven and half hours later. Notably, the results obtained during this 8-hour experiment could be shown in real time, and the data could be monitored offline from the back end.

## 4. Discussion

This study adopted unique and novel methods to detect mental fatigue and used muscle contraction frequency and hand grip strength as indicators of fatigue. Unlike other common methods of mental fatigue detection, which mainly focus on the facial expression, the method proposed herein emphasizes body detection. Numerous studies have explored the effectiveness of using face recognition to measure mental fatigue and have generated excellent results. However, by incorporating physiological signals in the process of fatigue identification, gradual variations in the respondents' mental fatigue statuses can be observed and the actual muscle fatigue status of on-duty security guards can be more accurately calculated. This system also supports monitoring the mental fatigue statuses of patrol guards at multiple sites. As long as the patrol area is moderate in size, data can be sent for offsite analysis using a distance Internet system. Furthermore, because one IP address is sufficient for the data transmission, related expenses are reduced.

In the present study, a mental fatigue monitoring experiment was designed to simulate the conditions of a patrol shift, and respondents were recruited to act as security guards and provide various physiological data. Notably, the respondents' maximum hand grip strength and median frequency of muscle contraction both decreased over time, indicating that muscle fatigue had developed. Specifically, the median EMG signal frequencies began to drop substantially at 4 and 5.5 h after the respondents' “shift” began, and their maximum grip strength decreased by 60%–70%. Although there are only two subjects involved in this study, the result is very similar with the previous studies [11, 17]. It may be considered as muscle fatigue if 20% decrease in median frequency was observed. In this study, there is 40% decrease after overnight patrol. It is a case-series study to examine the novel idea to examine patrolling security guards fatigue with physiological features.

Overall, these results indicate that the proposed system can detect early stage mental fatigue. Although limited data have been sampled, this experiment is presented as a case-series study; the results based on the two respondents should be utilized to conduct a large-scale testing of the proposed system.

## 5. Conclusions

In this study, a system that can detect the mental fatigue level of patrolling security guards was designed, integrating a camera with a thermometer, hand dynamometer, and electromyography. This system can determine whether an on-duty security guard signs in punctually and whether he or she is fit to stay on duty based on their physical conditions. This system is useful to avoid further burnout and stress in the workplace.
